# Supplementary material for: The effect of health-promoting leadership of nursing managers on the work withdrawal behaviors and psychological safety of nurses
Source: PLoS One. 2025 Apr 8;20(4):e0321131. doi: 10.1371/journal.pone.0321131 (PMC11978039; doi:10.1371/journal.pone.0321131)
Supplement: Attachment — Submitted filename: RES. to. Reviewer Comments-1.docx [file pone.0321131.s001.docx]

**Response to Comments from Editor and Reviewers**

***Dear Editor***

The authors would like to thank reviewers and editor for careful review of our manuscript and providing us with their comments and suggestion to improve the quality of the manuscript. The following responses have been prepared to address all of the reviewers’ comments in a point -by-point fashion. Please note that changes are in Track changes or blue marked in the revised manuscript.

***Dear Reviewers***

***Dear Reviewer 1***

*Comment 1: SEM model should be more detailed, such as predecessors if the study of SEM model* **Response to Comment 1:** Thank you for your comment . Dear reviewer, due to the length of the “introduction part”, explanations related to SEM model were added in the Methods section, Data Analysis, page 10.

*Comment 2: RESULTS：There should be no such thing as “Zero”. It should be precise to the number of women. The number of women is not shown in Table 1. Please complete.*

**Response to Comment 2:** Thanks for your careful review. Dear reviewer we did not have a variable for women in Table 1. In fact, it turned out that Table 1, which was related to demographic information, was accidentally deleted. Based on your comment, the table was added back to the revised file.

*Comment 3. The indicators in the table 2. do not meet the requirements, please check*

**Response to Comment 3:** We appreciate the reviewer’ insightful comments and helpful suggestions. We rechecked the table and only in the first part, due to translation errors, the number was typed incorrectly, which we corrected.

***Dear Reviewer 2***

***Comment 1. General Comments***

*The manuscript demonstrates substantial effort and commitment to exploring an important area in organizational health research. However, the translation to English has introduced some challenges. Specifically:*

*1. Language and Terminology:*

*- The manuscript contains instances of imprecise terminology, phrasing, and redundancy that could potentially mislead readers. For example, terms such as "proven" are not typically used in scientific writing when describing findings from correlational studies. Phrases like "associated with" or "linked to" would be more appropriate in this context.*

*- The language in the manuscript could benefit from significant refinement to improve readability and avoid ambiguity. I strongly recommend seeking the assistance of a professional English editor familiar with scientific writing to ensure clarity and accuracy.*

**Response to Comment 1:** Thank you for your comment. Dear reviewer we used the services of translation agencies to translate the article, and a native editing certificate was attached to the article. However, this time a native Persian-speaking professor also edited the article for content, grammar, and academic accuracy, and the changes are visible in the file with a track changes.

***Comment 2. Measurement Instruments:***

*- The manuscript uses several instruments, but more detail is needed to enhance transparency:*

*- Health Promoting Leadership Questionnaire: Providing a brief description of this instrument, including sample questions, would help readers better understand the construct being measured. The authors should clarify whether they used the employee-perception component, the leader self-assessment component, or both. Additionally, it would be helpful to specify whom the employees were asked to consider when completing the questionnaire (e.g., head nurse, nurse manager) as different organizations use different nursing leadership structures.*

*- Work Withdrawal Behaviors Questionnaire: Providing a brief description of this instrument, including sample questions, would help readers better understand the construct being measured.*

*- Edmonson’s Psychological Safety Questionnaire: Similarly, a brief discussion of this instrument and a few example questions would clarify how psychological safety was operationalized.*

**Response to Comment 2:** Thank you for your comment. All items requested by the reviewer were added to the Method section. Please refer to pages 7-9.

***3. Findings and Interpretation:***

*- Some of the conclusions drawn in the manuscript are presented in a causal manner, which is not supported by the study’s design. For example, in the Abstract, the statement: "The results of the present study showed that leaders who put health-promoting leadership on their agenda witnessed positive organizational outcomes, including improved psychological safety and fewer withdrawal behaviors" implies causation which the authors extend to organizational outcomes -- which are not examined as an outcome in this study. As this study appears to be descriptive and correlational, it is more appropriate to describe relationships as associations rather than causal effects. This issue may stem from translation challenges rather than conceptual errors, but it requires correction for accuracy.*

**Response to Comment 3:** We appreciate the reviewer’ insightful comments and helpful suggestions. The abstract was modified based on the suggestions.

***Suggestions for Revision***

*1. Revise language and terminology throughout the manuscript for precision and scientific rigor. Focus on using phrasing consistent with correlational findings.*

*2. Provide more detail on the methodology, including the rationale for the effect size threshold, sampling strategy, and the use of measurement instruments.*

*3. Clarify how the Health Promoting Leadership Questionnaire was applied, and specify the target leaders employees were asked to evaluate.*

*4. Include sample items from the Work Withdrawal Behaviors Questionnaire and Edmonson’s Psychological Safety Questionnaire to enhance understanding of the constructs.*

*5. Ensure that all findings and conclusions are framed in a way that reflects the study’s correlational nature.*

*6. Ensure that all findings and conclusions are not extended beyond the study's scope to include outcomes that were not measured (e.g. "positive organizational outcomes,"*

**Response to Comment:** Thank you for your time and effort**.** We appreciate the reviewer’ insightful comments and helpful suggestions. We have made all changes suggested by the Reviewer.
